# Intelligence as a Developing Function: A Neuroconstructivist Approach

**DOI:** 10.3390/jintelligence5020018

**Published:** 2017-04-29

**Authors:** Luca Rinaldi, Annette Karmiloff-Smith

**Affiliations:** 1 Department of Brain and Behavioural Sciences, University of Pavia, Pavia 27100, Italy; 2 Milan Center for Neuroscience, Milano 20126, Italy; 3 Centre for Brain and Cognitive Development, Birkbeck, London WC1E 7HX, UK; a.karmiloff-smith@bbk.ac.uk

**Keywords:** intelligence, individual differences, development, neuroconstructivism, emergent structure, developmental trajectory

## Abstract

The concept of intelligence encompasses the mental abilities necessary to survival and advancement in any environmental context. Attempts to grasp this multifaceted concept through a relatively simple operationalization have fostered the notion that individual differences in intelligence can often be expressed by a single score. This predominant position has contributed to expect intelligence profiles to remain substantially stable over the course of ontogenetic development and, more generally, across the life-span. These tendencies, however, are biased by the still limited number of empirical reports taking a developmental perspective on intelligence. Viewing intelligence as a dynamic concept, indeed, implies the need to identify full developmental trajectories, to assess how genes, brain, cognition, and environment interact with each other. In the present paper, we describe how a neuroconstructivist approach better explains why intelligence can rise or fall over development, as a result of a fluctuating interaction between the developing system itself and the environmental factors involved at different times across ontogenesis.

## 1. Introduction

“Intelligence constitutes the state of equilibrium towards which tend all the successive adaptations of a sensori-motor and cognitive nature, as well as all assimilatory and accommodatory interactions between the organism and the environment.”

*Jean Piaget* [1] *(p. 10) 1*

Attempts to grasp the construct of intelligence have quickly raised over the last century, making this concept one of the most extensively debated in the whole history of modern psychology. Despite its long odyssey through the psychological and neuroscience communities, however, scholars are still looking for an exact definition of intelligence and for its suitable theoretical scaffold [2,3]. Importantly, while our understanding of adult intelligence has rapidly progressed, our grasp of its developmental counterpart has moved along a slightly different path, and is still under way [4,5]. The reasons for this depend on a host of factors that make difficult capturing the dynamic nature of intelligence in childhood and adolescence.

This paper stems from the need to better capture the developmental essence of intelligence. After discussing the major achievements in the field, the main issues of debate, and the still open questions, we will propose a neuroconstructivist approach to the development of human intelligence. Neuroconstructivism, indeed, depicts development as a trajectory modulated by multiple interacting biological and environmental constraints [6,7,8]. Intelligence is possibly the ideal construct to be explained within such a neuroconstructivist viewpoint. Alas, it is outside our purpose to provide an exhaustive review of the most influential forms of theorizing and testing, as no single article, chapter or book can summarize theory and research on intelligence. Rather, we wittingly consider this paper as an effort to gather a unique glimpse into child and adolescent intelligence.

In Section 2, we will briefly introduce some general foundations about intelligence theorizing and testing. In Section 3, we will move on to maintain that intelligence in development is less stable than often believed, by providing a systematic review of studies examining the alleged stability of cognitive abilities across development. In particular, we will review three independent lines of evidence to challenge the classic view of intelligence as a stable trait. These denote, respectively, the difficulty in obtaining reliable intelligence assessment, the non-negligible fluctuations of intelligence scores and their related neural changes, which occur to a greater extent in the early stages of life compared to adolescence and adulthood. Based on the evidence reviewed, in Section 4 we will argue how a neuroconstructivist approach can more parsimoniously describe the dynamic nature of intelligence during development than theoretical models grounded on the principle of intelligence stability. Specifically, we will define a common set of developmental principles and mechanisms that operate and interact at different levels (i.e., genetic, cellular, neural, behavioral and environmental) and whose effects on cognitive abilities are being increasingly documented. Indeed, the literature is currently scarce on theoretical perspectives that simultaneously consider all the different constraints affecting the development of intelligence. Finally, in Section 5, we will illustrate the benefits of this approach on public policies, especially considering that intelligence scores are a good predictor of many life outcomes.

## 2. Intelligence: General Caveats 

Definitions of intelligence and psychometric tests for measuring cognitive abilities have mushroomed during the past century. In this section, we first provide some general definitions of this extensively debated concept and then we describe the basic proprieties beyond intelligence testing to break the ground for the core issue of this paper, i.e., the development of human intelligence.

### 2.1. On Some Definitions of Intelligence

Achieving consensus among influential experts about the definition of intelligence has always been a difficult task. A comprehensive definition was expressed by the American Psychological Association in 1995 [9]: “*Individuals differ from one another in their ability to understand complex ideas, to adapt effectively to the environment, to learn from experience, to engage in various forms of reasoning, to overcome obstacles by taking thought. Although these individual differences can be substantial, they are never entirely consistent: a given person’s intellectual performance will vary on different occasions, in different domains, as judged by different criteria. Concepts of ‘intelligence’ are attempts to clarify and organize this complex set of phenomena*”.

Intelligence, thus, encompasses the mental abilities essential for survival and advancement in any environmental context. The truth, however, is that behind an apparently simple definition of intelligence lies the complexity of our cognitive architecture to make effective adaptation possible. A number of cognitive processes, such as perception, learning, memory, reasoning and problem solving are indeed necessary in the right combination to learn, understand and deal with new situations [10]. Regardless of this complexity, researchers have found various ways to measure it. In clinical and research contexts, intelligence has almost always been computed from explicit behavior. Typically, an individual has to respond quickly or accurately to a given stimulus or question, presented among different possibilities. The individual’s performance is then rated for speed, accuracy or more subtle aspects, such as learning. Individuals generally differ substantially in their performance, and those who perform well on one test tend to do well on many other related tests. All intelligence tests, ranging from unitary tasks to multi-faceted tasks, tend to generate a strong general factor called as “Spearman’s *g*” or “general intelligence” (i.e., the first component of a factor analysis) [11,12]. This score can be conceived as a single summary measure of cognitive ability and it is made up of a small number of non-independent subfactors that represent more specific abilities.

Many decades after the introduction of Spearman’s *g*, Cattell further specified this single summary measure of cognitive ability, differentiating between fluid intelligence (*g*F) and crystallized intelligence (*g*C) [13]. In particular, *g*F includes a series of abilities, such as reasoning, perceptual knowledge, and novel problem-solving ability [14] and, from an empirical standpoint, it is strongly associated with working memory and with *g* [15,16]. On the contrary, *g*C refers to overlearned skills and static knowledge acquired through educational experiences over lifetime, such as vocabulary, and it is more resistant than *g*F to the effects of brain insults or other intervening events in the course of normal development [17].

In contrast to the *g* factor, intelligence quotient (IQ) has been conceived as a composite measure that derives from performance on a variety of cognitive tasks, including working memory, verbal comprehension, and visuospatial processing [18]. Even though terms like *IQ*, *g* and *mental ability* have been distinguished on some occasions, in this paper, we will rather use these expressions interchangeably to generally describe the concept of intelligence [3].

### 2.2. The Importance of Intelligence Testing

One of the greatest achievements in the history of psychology and, at the same time, one of its most controversial issues, pertains to the measurement of intelligence. Different critiques, indeed, have been advanced, especially complaining that no single test can measure the complexity of human intelligence and that this measurement is inevitably affected by cultural factors. Although these critiques have proper validity, there is no doubt that the measurement of intelligence has enormous practical value, as it is a soundly good predictor of educational attainment, performance at work, and many other aspects of success in life [3].

Individual differences in intelligence are nowadays typically measured by means of psychometric tests, which cover a range of various cognitive domains such as problem solving, executive function, memory, processing speed, verbal and spatial abilities. Intelligence tests should show marked individual differences, which are considered to be quite stable in rank order throughout development and even over long time spans [19]. Intelligence differences in the population nearly follow a normal distribution, with the only exception of the slight excess at the lower end of the distribution due to individuals with severe disorders having poor cognitive abilities [20]. Of particular relevance is the fact that the coupling between intelligence testing and psychological theory has always been moderately weak. Individual tests of intelligence are, in fact, relatively uninfluenced by theory and this gap is even more accentuated when it comes to development [5], as discussed in the next section. For instance, developmental perspectives on intelligence hark back to Jean Piaget, even though his theory on cognitive development has been only barely used as a model for intelligence research [4].

## 3. Intelligence across Development: A Dynamic Concept

Section 3 is devoted to introducing the dynamic essence of general cognitive ability over development. Three types of evidence are reviewed to support the hypothesis that intelligence profiles can rise or fall over development, thus challenging the idea that cognitive abilities are fixed and static. First, we will discuss the reasons that make it difficult obtaining stable, reliable and valid intelligence assessment early in life. Second, we will review the evidence pointing to substantial fluctuations of intelligence scores over development. Third, we will pinpoint that such fluctuations are accompanied by analogous neural changes, thus providing validity to the described behavioral changes.

### 3.1. Intelligence Testing across Development

Starting from early primary school years, researchers and clinicians assess intelligence through standardized test batteries (i.e., IQ tests), as these tests are intended to be suitable for a wide age range. The Wechsler Intelligence Scale for Children (WISC, which provides both Verbal and Non-Verbal IQ scores) [21] and the Raven’s Progressive Matrices (i.e., both the Colored and Standard versions) [22] are among the psychological tests most commonly used for the 6–17-year age range [23], and are taken as a good index of general intelligence. The use of a “g” score in developmental testing, however, may have some important limitations. For instance, a child may receive a relatively low or high composite score, yet presenting highly dissociated skills in more specific domains.

Intelligence test instruments available for use in the preschool age differ more consistently in terms of their normative data, the age range for which they are appropriate, the factor analytic model of intelligence they rely on, and the types of behavior they measure. Furthermore, infant and preschooler measures, which are typically associated with the age range of 1–6 years, differ from other intelligence tests in scope. Infant measures are indeed generally multidimensional, as they frequently assess cognitive and motor domains simultaneously. Consequently, until some years ago, the prevailing opinion was that a reliable measurement of general intelligence was possible only once a child reached five years of age [24]. In more recent years, however, instruments aimed at assessing intelligence in the preschool age have improved significantly [5]. Common tests for this age range are the Bayley Scales of Infant and Toddler Development [25], the Wechsler Preschool and Primary Intelligence Scale [26].

In interpreting an intelligence measurement it is fundamental to bear in mind that the inferred score does inevitably include a certain amount of error. This is called the standard error of measurement and it can be quantified as an estimate of a “true” score based on observed scores [27]. In normal intelligence testing, the standard error of measuring is often larger for higher scores than for lower scores, and critically, it tends to be greater in the early stages of development [5,27]. In fact, preschoolers have often very distinct developmental and maturational trajectories, as reflected by the huge variability in the age of acquisition of new basic skills. This obviously makes the assessment of a young child less likely to be stable compared to the one of an older child.

Many other aspects, such as the circumstances that complicate any attempt to assess a very young child may represent another source of error of measurement [5]. Conducting an assessment with young children is indeed very complex. The examiner should create interest and motivation and keep the child on task, but at the same time should try to follow standardized procedures [4]. Realistically, though, the assessment of children younger than 24 months frequently implies some modifications in the standard procedure, such as for example in the assessment order. Moreover, although caretakers may even administer some of the test items to preschoolers and thus further undermine test reliability, they should not be present during the evaluation of older children [4].

Additional challenges in obtaining reliable and valid assessment at the early stages of development come from the dyadic interaction during testing. Examiner proficiency in building relationship, eliciting optimal cooperation, maintaining target behavior, and recognizing subtle qualitative aspects of behavior can indeed influence the validity of the testing [5]. Similarly, performance on intelligence tests is also biased by the child’s comfort in separating from the parent, his/her compliance with the examiner, temperament, motivation, health, sleep and nutritional patterns [28]. Parental attitudes and socioeconomic status may further contribute to the child engagement and, consequently, to determining the relative intelligence score [5]. However, the idea that intelligence tests underestimate the cognitive abilities of children from non-supportive environments has so far received conflicting support [29].

Together, the difficulties described above in the assessment of intelligence during development, along with the different individual maturational trajectories, may largely undermine not only the validity, but also the stability of the measurement itself, especially in infancy and early childhood.

### 3.2. Intelligence Fluctuations across Development

In most cultures, intelligence is considered as a stable trait of an individual. This common view conceives cognitive abilities as static, with a level of intelligence that should remain relatively stable over time, as evidenced by the fact that IQ measurements made at different points in an individual’s life tend to correlate well [19,20]. Efforts to challenge this view, however, are not lacking. For instance, Valsiner [30] contended that intelligence theories should move from being primarily internal or static in nature to being dynamic and expressed as a sign of relationship with the context. Strong correlations over time can indeed hide non-negligible individual variation. Over 50% of the variation remains unexplained when two intelligence scores, measured at different times, show a correlation coefficient of 0.7.

In the same vein, two-component theories of intellectual development (e.g., [14,31]) suggest an alternative view to a static model of intelligence, by proposing that different components may dissociate in the direction of their development. Indeed, *g*F tends to peak early in life and to show gradual age-related declines starting from adulthood. On the contrary, *g*C, which is believed to reflect cultural assimilation, generally continues to increase throughout life [32,33] (please see Box 1). In the paragraphs below, we will provide further support to the view that intelligence is particularly flexible over development, by discussing three apparently independent lines of evidence supporting this hypothesis.

**Figure 1:**
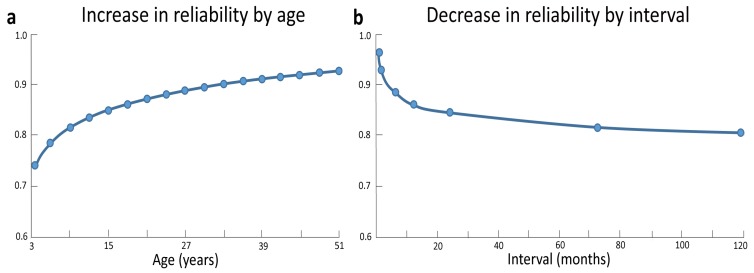
Test-retest reliability data gathered from 34 separate studies on common IQ tests (the Standford-Binet, the WISC, the WISC-R, the WAIS, and the WAIS-R) indicate a drastic increase in reliability with age, with less stable scores in childhood and early adolescence compared to late adulthood (**a**). The interval between testing was another correlate of stability, with a drop in reliability as interval increases (**b**). Adapted and reprinted with permission from John Wiley and Sons: Journal of Clinical Psychology [40] © (1989).

A first aspect concerns the stability of intelligence over time. There is broad agreement that the stability of intelligence depends on the specific age range considered (e.g., [34]). Most studies did not find good longitudinal consistency in intelligence scores between preschool years and later stages in development (e.g., [35,36]), with cognitive abilities becoming relatively stable only from childhood onwards (e.g., [37,38,39]). By reviewing a number of studies in the literature, Schuerger and Witt [40] found that in about 13% of the 6-years-old scores changed at least of one standard deviation (i.e., a huge change, considering that standard intelligence tests have a mean of 100 and a standard deviation of 15) and the same was true in 7% of the 30-years-old. Indeed, a drastic increase in test-retest reliability occurs as a function of age, with less stable scores in childhood and adolescence compared to adulthood (see Figure 1a). Along these lines, a recent study revealed that, despite a good test–retest correlation across a two-year interval (*r* = .81 for Full Scale IQ), 25% of typically developing children and adolescents showed changes of nine points or more across this interval [41]. Similarly, correlations between cognitive abilities at the age of 17 and measures taken during preschool years increased from *r* = .16 at the age of 1 year to *r* = .44 at the age of 3.5 years, with coefficients becoming increasingly stronger at the time of school enrolment (six years; *r* = .67) and later on during school years (eight years; *r* = .77) [42,43], and especially from the age of 11 years onward [44]. It is worth noting, however, that even during late childhood significant increases and decreases in individual levels of IQ are not uncommon. These fluctuations have often been ascribed to measurement error [45]. However, some of these fluctuations can also represent true changes in cognitive abilities, as evidenced by parallel changes at the brain level (see the next section).

A second aspect concerns the stability of intelligence as a function of measurement interval. Indeed, the longer the interval between two intelligence measurements the greater the instability [40] (see Figure 1b). Accordingly, stability coefficients of WISC IQ scores drastically decline from the .80–.90 range in short-term test-retest investigations to the .50–.90 range in longer retest intervals (e.g., three years or more) [34,46,47].

A third and final aspect pertains to the stability of intelligence as a function of IQ level (i.e., high, average, and low). In particular, it has been repeatedly shown that high scores in young children are less stable than low scores. For instance, in a sample of 4-year-old children who were followed up until the age of 23, those with initial low IQ scores showed overall more stability over time than those with average and high IQ scores (e.g., [43,48,49]). However, intelligence fluctuations can occur even in children with low IQ. That is, children with low intelligence scores (i.e., <85) can subsequently reach scores above 120 at the age of 17, thus again suggesting the occurrence of drastic changes, at least at the individual level, during development [43].

Box 1 **The differentiation-dedifferentiation hypothesis.**

The fact that the level of cognitive performance varies so dramatically with age has led some theorists to propose a gradual differentiation of general ability into fairly distinct aptitudes over the life span [50,51,52]. In particular, according to the *differentiation-dedifferentiation hypothesis* (e.g., [53]) intellectual skills (i.e., such as *g*F and *g*C) are rather undifferentiated in childhood. However, the accumulation of environmental and non-cognitive (e.g., interest, motivation) influences over time would prompt independent trajectories between *g*F and *g*C. Finally, with advancing age, these abilities would become undifferentiated again (i.e., *g*F once again more closely correlated with *g*C) (but see [54]).

It is worth specifying, however, that a number of behavioral genetic investigations predicts patterns inconsistent with this hypothesis (see for a discussion [54]). According to these studies, the proportion of individual differences attributed to genetic sources increases across childhood and adult lifespan, as the individual selects environments that are compatible with their ability levels, thus amplifying prior differences (e.g., [55,56]).

### 3.3. Neural Changes Associated with Intelligence across Development

Various neurobiological markers have been associated with individual differences in IQ. These include total brain volume [57], cortical thickness [58,59], white matter tract integrity [60], and more efficient brain activity both during task performance [61] and at rest [62]. Similar findings have been reported also in the developing brain. For instance, IQ is positively correlated with total cerebral volume in children [63], and adolescents [64], as well as with a thicker cortex [65,66], and this association is partly of genetic origin [2]. Further, subcortical grey matter contributes to variance in IQ, although to a lesser extent than cortical grey matter [67].

Importantly, the wide-spread use of modern neuroimaging has allowed to test whether unexpected longitudinal fluctuations in IQ may be related to brain development. In fact, despite the human cortex mostly grows during the prenatal period [68], recent findings suggest that postnatal structural brain development is substantially plastic [69,70]. Interestingly, this plasticity seems to be related to changes in IQ scores. In a seminal longitudinal investigation, Shaw and colleagues [67] showed that patterns of correlation between intelligence and brain structure (e.g., cortical thickness) vary as a function of participant’s age. In particular, developmental trajectories of cortical thickness (i.e., increase and subsequent thinning) appear delayed in more intelligent children [67]. These findings corroborate the evidence of fluctuations in IQ behavioral scores described above and suggest that cognitive abilities may be associated more to the magnitude and timing of developmental changes in brain structure than to brain structure per se [71].

**Figure 2:**
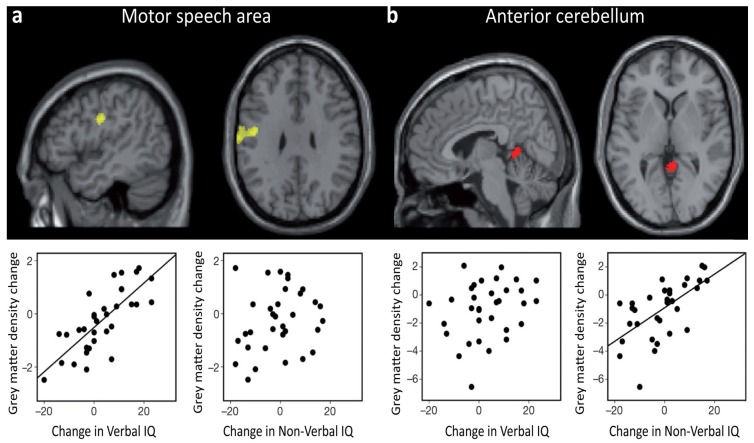
In the study by Ramsden and colleagues [72], 33 neurologically normal adolescents had structural and functional brain scans, along with an IQ measurement (Wechsler Adult Intelligence Scale III and WISC III), at two different times (Time 1: 12–16  years old; Time 2: 15–20  years old). Results showed that changes in Verbal IQ, observed between the two time points, were positively correlated with changes in grey matter density (and volume) in a region of the left motor cortex, which is activated by the articulation of speech (**a**). In striking contrast, changes in Non-Verbal IQ, observed between the two time points, were positively correlated with grey matter density in the anterior cerebellum, which is associated with motor movements of the hand (**b**). Adapted and reprinted with permission from Macmillan Publishers Ltd: Nature [72] © (2011).

Another neuroimaging study illustrates the dynamic essence of intelligence-brain relations. By combining structural and functional imaging, Ramsden and colleagues [72] found that in adolescents verbal IQ fluctuations were accompanied by grey matter changes in a region that was activated by speech, whereas non-verbal IQ fluctuations were accompanied by grey matter changes in a region that was activated by finger movements. This speaks in favor of considerable effects of brain plasticity associated to IQ during the teenage years (see Figure 2). The idea that changes in intelligence measures across development can reflect meaningful changes in general cognitive abilities and in their neuroanatomical substrate was further supported by a recent study based on a sizeable sample of children and adolescents [73].

### 3.4. Following Developmental Trajectories

The evidence reviewed before challenges the classic view of intelligence as a stable trait, by highlighting that IQ levels undergo substantial fluctuations over development. In addition to this, it is worth specifying that recent proposals suggest that also the composition of the central core of intelligence is not stable, but rather changes and develops through the years (see Box 2). The main challenge for those developmental psychologists who aim to reach a full grasp of intelligence during development is therefore to integrate individual observations into a developmental trajectory, and to consider the multiple sources that are influencing it, in the attempt to identify possible mechanisms driving developmental change. In this sense, a broad theoretical framework is needed to better understand intelligence over development and to account for its dynamic essence at various levels of analysis, possibly including biological, environmental and behavioral correlates of IQ.

Box 2 **How does intelligence progress over developmental time?**

Recent theorizations suggest that intellectual development is characterized by a progressive specialization in children’s representational capacity. In particular, three fundamental processes would sustain intellectual functioning across development by continuously generating representations of increasing inclusiveness and resolution: *abstraction*, *alignment*, and *cognizance* (AACog) [74,75,76]. First, *abstraction* extracts similarities between patterns of information, through mechanisms that may vary in development. Next, *alignment* allows to group and relate representations in terms of their possible similarities. Finally, *cognizance* is the component of consciousness focusing on the mind itself and protracts experience from past to present for *abstraction* and *alignment*. In both theoretical and empirical terms, AACog is conceived as a common core similar to *g*F, with different levels of IQ that would correspond to the different types of representations and problem-solving that a child can master [76,77]. AACog operates from the very early stages of infancy, although the relative contribution of each process may vary as a function of age, and progresses through four major developmental cycles, each comprising two phases [74,78,79,80,81]. Specifically, in a first phase, new representations emerge and next they are aligned, with each cycle terminating with insights about the cycle’s representations [82]. In this way, the type of representations that the child can master, their inter-relations and their actual awareness change with age. Importantly, conceptual development is self-propagated because AACog continuously generates new mental content expressed in representations of increasing inclusiveness and resolution [74].

This model has also been shown to capture the dynamical relations between three principal component of mental functioning (attention control, executive control, working memory) and *g*F. Results of different studies, indeed, demonstrated that the relationship between these components and *g*F changes periodically with age [83,84,85]. That is, in the first phase of each cycle the relations between scores of processing speed (i.e., indexes of attention control and executive control) and *g*F tend to be high, whereas the relations between working memory and *g*F tend to be low. In striking contrast, these relations become inverted in the second phase of each cycle. Together, this evidence indicates that the developmental structure of intelligence systematically changes across development.

## 4. A Neuroconstructivist Approach to Intelligence

In this section, we will argue that neuroconstructivism [6,7,86], which emphasizes the interrelation between brain and cognitive development, may provide a parsimonious developmental framework to account for the developmental essence of intelligence. In fact, the adoption of a neuroconstructivist approach is particularly relevant for the current state of the art in intelligence research, whereby multiple sources of constraints have been already shown to influence intellectual functioning across developmental time. However, insofar these constraints have not been connected together by a single theoretical framework that emphasizes the dynamic and multilevel nature of this concept. On these grounds, here we attempt to incorporate for the first time these multiple levels into a unified model by taking a developmental perspective.

We begin by arguing that, within this theoretical framework, we conceive intellectual development as a progressive increase in the efficiency of representations through experience-dependent processes. In development, indeed, new representations can progress only on the basis of previous and simpler representations. Apparently, our view is thus similar in many respects with other theories described before (see Box 2), whereby intelligence changes with development at various levels, including the nature of representations and the refinement of their manipulation [77]. It is, nevertheless, unique in four different respects.

First, representations are here explicitly conceived as neural activation patterns that sustain adaptive behavior in the environment [87]. Within this framework, the progress through representational efficiency takes place in the brain by means of a progressive refinement of cortical structures. Consequently, understanding the development of intelligence implies an understanding of how the neural substrates supporting the progress of mental representations are shaped. Variation in brain structure and function, indeed, can be used to discriminate the intellectual functioning of different individuals, as reviewed before [20]. Further, a series of neuroimaging studies have shown a consistent negative relationship between brain activation and intelligence (see for a review [88]). Specifically, brains of intelligent individuals are more functionally efficient in that they use fewer neural resources (i.e., less energy consuming) when performing cognitive tasks compared to brains of less intelligent individuals (the so-called “neural efficiency hypothesis” [61]; but see [89]). The need here is therefore to identify those factors that lead to the development of efficient neural activation patterns promoting adaptive behavior.

Second, and strictly related to the above need, we conceive the development of these increasingly complex representations as profoundly constrained by multiple factors, which are both intrinsic and extrinsic to the developing organism. By recognizing the role of multiple constraints, this framework integrates different views of brain and cognitive development, including: (a) probabilistic epigenesis, which underscores the interactions between experience and gene expression [90]; (b) neural constructivism, which emphasizes the role of experience on development of small-scale neural structures [91]; (c) the “interactive specialization” view of brain development, according to which the shift from distributed to more localized processing would be due to activity-dependent interactions between brain regions [92]; (d) the embodiment view, which maintains that bodily states are necessary for cognition, especially during development [93,94]; and (e) the ensocialment view, which recognizes a crucial role of social environment for the developing child (e.g., [24,95,96]). These five views thus encompass different levels of constraints (i.e., genetic, cellular, neural, behavioral and environmental) that would influence the development of intellectual functioning [6].

Third, we maintain that the emergence of mental representations is not influenced by each of these levels separately. Rather, by taking a neuroconstructivist perspective, we assume a strict interdependency between these levels [97]. A principle of *context dependence* thus operates on all levels of analysis. In fact, the modeling of neural structures is highly dependent on the context in which these structures develop. Representations in the brain do not develop in isolation. Rather, the constraints that shape the developing neural system necessarily alter the actual context in which the individual develop, affecting consequently the developmental trajectory itself and the specific outcome that is measured [8,98]. Thus, a change at one level has the potentiality to affect all the others.

Fourth, and finally, this approach maintains that the constraints on mental representations can be systematically varied across development. The rise and fall of intelligence profiles over time at both behavioral and neural levels can therefore be explained by the developmental variations in the constraints affecting intellectual functioning. For instance, gene expression in the brain changes as a function of environmental experience over developmental time. For this very reason, it is crucial to follow neural activation patterns that sustain adaptive behavior at multiple levels of analysis over time. Importantly, adulthood is here viewed as a more stable state along the developmental trajectory [20], as the constraints operate in a greater extent during the early phases of life. Consequently, intelligence profiles are expected to be more stable in adulthood compared to infancy and early childhood, in line with the evidence reviewed above.

To sum up, intelligence is here conceived as an emergent propriety originating from multiple interactions between the constraints imposed by genes, brain, behavior, cognition and environment. For this very reason, we suggest that it is only by simultaneously considering all these constraints that we can better explain why intelligence can rise or fall over development. In the following paragraphs, we make an explicit attempt to describe how the different levels of constraints shape the development of human intelligence and, specifically, how they relate to neural representation and behavioral outcomes. In particular, we present below a set of interpretations, along with initial available empirical data, which provide support to a neuroconstructivist view on intelligence development.

### 4.1. Probabilistic Epigenesis

Until the past decade, development was mainly thought as a predetermined expression of genes [99]. The view of a genetic blueprint for development has nevertheless been recently challenged, by recognizing the primary role of environmental and behavioral influences on the developing organism. The view of epigenesis has shifted from deterministic, unidirectional, and under tight genetic control, to probabilistic, bidirectional, and under broad genetic control [90]. In particular, this probabilistic epigenetic view of development maintains the existence of pervasive dynamic interactions between genes, neural activity, and the physical and social environments of the developing child [100,101].

Here we summarize findings that support the view that intelligence develops along a similar probabilistic epigenetic route. Substantial empirical work suggests that genes may work via the environment to shape IQ measures [102]. The heritability of intelligence—i.e., the proportion of observed variance that can be ascribed to genetic factors—in any naturally occurring population is in fact neither zero nor one. The acknowledgment of both genetic and environmental influences has increased the study of their interplay in modulating (i.e., in terms of moderation and mediation effects) the development of intelligence [103]. Indeed, the relationship between a specific gene and intelligence is often very indirect and complex.

Unfortunately, research has not yet identified a precise genetic locus that contribute to normal variation in intelligence scores [104]. Intelligence is a complex behavioral trait and, as such, is highly polygenic, in the sense that many genes contribute to individual variation [105]. This is not surprising, given that very different cognitive abilities, such as spatial ability, vocabulary, executive function and memory, contribute in defining intelligence scores [106]. However, it is now time for a developmental molecular genetics of cognition that may take advantage of recent advances in technology and technique (such as genome-wide association study, genome-wide complex-trait analysis and DNA resequencing studies). Insofar, only few efforts have been made in this direction (e.g., [107,108]).

The probabilistic epigenetic view has also contributed to a better understanding of the shift from distributed to more localized processing that would occur over many months, or even years, across development [109]. Indeed, gene expression in the brain would change from initial widespread gradients across the cortex [110], restricting expression to more specialized cognitive-level circuits only progressively in time. This shift may be reflected as well in the development of intelligence. The heritability of IQ scores seem to increase over the course of development [111,112], from 20% in infancy to 40% in adolescence, and to 60% in adulthood [103]. Importantly, this pattern is modulated by IQ level, as individuals with high IQ show reduced heritability in adolescence (i.e., resembling younger children), a tendency in line with the view of a an extended sensitive period for intellectual development in more-intelligent individuals [113]. Overall, this speaks in favor of more constant genetic influences on cognition in adolescence and adulthood compared to the first decade of life [114], a pattern elegantly accounted by the “genetic amplification” hypothesis [115,116]. According to this proposal, small genetic differences would be exaggerated as children select, modify and create environments correlated with their genetic propensities [103]. A proposal that put into the foreground genotype-environmental interactions views of intellectual functioning (but see for a discussion [117]).

### 4.2. Neural Constructivism 

The term “neural constructivism” is opposed to selectionist theories, as it implies that our neural architecture is extensively shaped by activity originating at various levels of the environment, ranging from the cellular to the social environment [118]. By adopting a constructive process of growth, neural constructivism conceives the protracted period of postnatal growth as essential in influencing the resulting domain specificity of the developing neocortex [86]. This influence of the environment on brain structure and function is well captured by research on environmental enrichment. Various studies have shown that environmental enrichment may exert a variety of effects on the brain, documented in several species of mammals [119]. For instance, brains in richer environments can show increases in cortical thickness and synaptic size and number, with stronger effects during neurodevelopment than in adulthood [119]. Together, this evidence speaks in favor of a circular loop between experience and the development of neural networks.

The same circular loop (or *context dependence*) describes the bidirectional relationship between environmental factors and neural structures subserving intellectual functioning. For instance, the relation between children’s socioeconomic status (SES) and individual differences in intelligence is one of those issues that often puts science in the public eye. The effect of environment on IQs and academic achievement scores of young children can indeed be significant, with children who grow up in poverty showing lower IQ scores [120,121]. This testifies to the wide impact of the socioeconomic environment on various neurocognitive domains, such as working memory, cognitive control and especially language and memory, which are highly interrelated with general intelligence [122].

**Figure 3:**
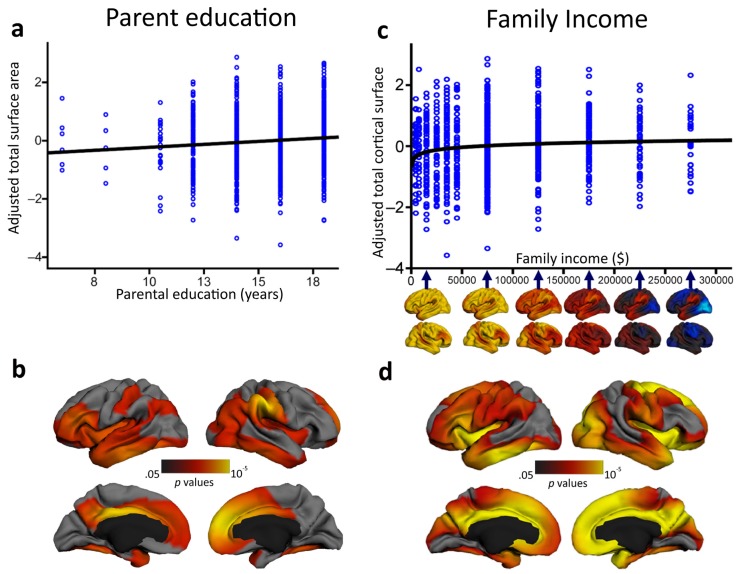
In a cohort of 1099 typically developing individuals between 3 and 20 years of age, parental education was found to be significantly associated with children's total cortical surface area (**a**) in different brain regions associated with language, reading, and various executive functions and spatial skills (**b**). Family income was significantly logarithmically associated with children's total cortical surface area (**c**) in widespread regions of children's bilateral frontal, temporal and parietal lobes (**d**). Adapted and reprinted with permission from Macmillan Publishers Ltd: Nature Neuroscience [127] © (2015).

However, does the socioeconomic environment have some effect on neural structures as well? Answering the skeptics: yes, it does. A number of studies have found a relationship between environmental factors and neurobehavioral functioning in children (e.g., [123,124,125]), with socioeconomic status that seems to moderate patterns of age-related cortical thinning [126]. In one of the largest studies to date to characterize associations between socioeconomic factors and children’s brain structure, parental education and family income accounted for individual variation in independent characteristics of brain structural development, independent of age, sex, and genetic ancestry [127]. Children who participated in the study underwent a standardized structural MRI protocol, provided saliva samples to assess genetic ancestry, and performed different behavioral tests of attention, working memory, vocabulary and reading. Parental education was found to be linearly associated with children’s total brain surface area (which is in turn associated with intelligence) over the course of childhood and adolescence. On the contrary, surface area mediated the link between family income and children’s performance on certain executive function tasks (see Figure 3). These findings add to the emerging literature indicating that SES relates to structural brain variation in the hippocampus [128], amygdala [128] and prefrontal cortex [129] (see for a review [130,131]). Although the study by Noble et al. [127] did not directly assess children’s intellectual functioning, intelligence has been associated with the trajectories of both cortical thickness and surface area during development (e.g., [67,73]). As such, these findings may suggest the existence of a strict interplay between intelligence, environmental factors and brain structure across development. However, it is worth specifying that Noble and coworkers’ investigation [127] was a non-experimental cross-sectional study and, as such, the driving link between SES and brain structure remains unclear. Indeed, this association may be mediated by the ability of more highly educated parents to earn higher incomes. Low-income parents face a much greater array of material hardships (housing, food, and medical costs) that can, in turn, exacerbate environmental risks and stress (e.g., [132]). In this sense, understanding the proximal interrelationships between brain functioning and more specific aspects beyond SES must be a priority for future research.

### 4.3. Interactive Specialization 

The interactive specialization view proposes that many cortical areas start out with poorly defined functions that become domain-specific over development, through a process of neuronal competition and gradual specialization, location and modularization of function [92,133,134]. This narrowing process is determined by the activity-dependent interactions between brain regions and causes, in turn, modifications of the intraregional connectivity [92]. As a consequence, cortical areas are much more highly interconnected in the infant brain than in the adult brain, with the ratio between white and gray matter that changes over development [135].

The development of intelligence seems to follow a similar process of neural specialization. First, a very recent study showed that preadolescent children with high scores in perceptual reasoning exhibited significantly greater global efficiency of structural brain networks [136]. This finding indicates that children with higher IQ scores have brain networks that are more highly integrated at both global and local levels (see also [137]). Organizational efficiency of white matter is indeed related to higher intelligence across the life-span [20]. Since brain development in childhood is associated with large-scale changes in synaptic connectivity, gray matter thickness and myelination, the extent of activation of this network may depend on age. Unfortunately, no study has so far explored whether the relationship between network's global efficiency and intelligence differs between childhood, adolescence and adulthood. Similarly, although it has been shown that individual differences in intelligence are associated with the functional connectivity between parietal and frontal brain regions in both adults [138] and young children (i.e., 6–8-years old) [139], it is not yet clear whether this association varies as a function of age.

Second, some groundbreaking studies indicate that the exploration of neurodevelopmental trajectories is crucial for understanding individual differences in intelligence [59,64]. Detecting changes in gray and white matter volume, as well as in cortical thickness, in the first years of life may therefore be essential for better grasping fluctuations in intelligence scores over development. Accordingly, it has been recently demonstrated that high expanding brain regions in both postnatal development and evolution, especially the anterior cingulate and some specific parts of the frontal cortex, tend to be related to intellectual functions in humans, as opposed to low expanding areas [140].

### 4.4. Embodiment

According to the embodiment view, proactive exploration and manipulation of the environment occupies a primary position in cognitive development [141]. This view is supported by increasing research showing that development depends on bodily states and situated action (e.g., [94]). In general, indeed, extensive amounts of learning proceeds through perception, action, and cognition, with the developing body that can serve as information filter to the brain. For instance, visual orienting is the main infants’ capacity of gathering information from the surrounding world for further study and learning [92]. Similarly, motor development in infancy relies on general and basic psychological functions necessary for survival, such as response selection, behavioral adaptation, and categorization.

It is not surprising, therefore, that the development of the attentional and sensory-motor systems has been linked to individual differences in general cognitive abilities. In fact, infant IQ measures heavily relay on the assessment of both attentional and motor domains. Back in the 80s, it was suggested that visual habituation and dishabituation may be promising candidates for predicting intelligence at later stages of life [142,143,144]. Visual habituation and dishabituation are dependent, at various degrees, on the gradual construction of a memory trace, the recall of information from memory, and the process of comparing the memory trace with the current visual input. The suggestion that attentional functioning in infants may relate to cognitive abilities was then corroborated by various studies documenting the predictive value of visual habituation and dishabituation on IQ later in life (see for a review [145]) as well as on academic achievement [146]. In particular, the overall weighted and normalized average correlation relating habituation/dishabituation to later IQ amounts to .37 (i.e., these measures can account for about 17% of the variance in later intelligence [145]). In line with this, a recent study from a longitudinal cohort of children tested twice in infancy (7 and 12 months), twice in the toddler years (24 and 36 months), and then again at 11 years, provides evidence of continuity for four domains of core cognitive abilities (i.e., attention, processing speed, memory and representational competence) and their relation to later IQ [147]. Importantly, the relationship between attentional functioning and intelligence would be mediated by environmental factors (see for a discussion, [148]). For instance, infants with caretakers who are less successful at attaining attention toward objects and events in their environment show less optimal cognitive and language outcomes during the second and third years of life, and lower intelligence scores at age 4, compared to infants with more encouraging caretakers (e.g., [149]).

**Figure 4:**
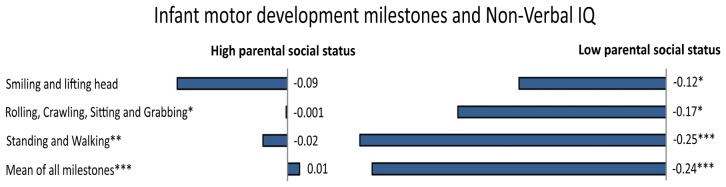
In a large Danish sample, the association between several specific infant motor developmental milestones and adult Non-Verbal IQ was moderated by parental social status (*p*-values immediately after the text, represented by the symbol *, indicate the level of significance for the interaction term with parental social class). In particular, stronger associations between milestone attainment and adult intelligence in the subsample from low social status families were observed (standardized regression for milestones predicting Non-Verbal IQ are reported). Adapted and reprinted with permission from Elsevier: Early Human Development [156] © (2015).

Infant motor development is also related to individual differences in cognitive abilities [150]. The rate of progression of infant motor development, especially age of walking, is associated with IQ at 64 months [151], 3 years [152] and at 6–11 years [153], and even with educational attainment [154] and adult brain structure [155]. That is, children who reach motor milestones earlier tend to have higher IQ scores later in life, compared to those who have a more delayed motor development. More specifically, some studies found that 4%–6% of the variance in IQ could be explained with respect to motor development (e.g., [150,152]), even when controlling for various confounding factors, such as mother’s cigarette consumption in the last trimester, gestational age, birth weight, and birth length [156]. Further, motor coordination in adolescence tends to be related to academic outcomes [157] and working memory [158]. Even in this case, environmental factors would play a critical role. Indeed, the relationship between early motor development and intelligence is stronger in infants of low parental social status than in those of high parental social status, with “sits without support” and “walks without support” that accounted for 5.3% and 9.2% of the variance in Non-Verbal IQ, respectively [156] (see Figure 4). This pattern is most likely to be explained by assuming that caretakers in high status families are generally aware of stimulating the child appropriately. Finally, the association between body mass index in preschool children and IQ also appears to be mediated by SES [159].

### 4.5. Ensocialment

The social environment in which a child develops is another fundamental level that constrains infants’ and children’s development. The relationship between socio-economic background and intelligence tests is a hotly debated issue, with both parents’ education [160] and family income [161] that have been related to IQ. Their possible interactive effects with the genetic, neural and cognitive counterparts have been widely described before. The timing of early developmental changes is in fact sensitive to exogenous influences, such as the quality of dyadic interaction and the level of stimulation from the environment [162]. In addition to this, parental attitudes also play a determinant role in engaging the child in those tasks that promote abilities linked to intelligence tests. For instance, childhood neglect, i.e., a failure to provide for or supervise one’s child, has a profound impact on IQ measures [163]. Furthermore, children with mothers depressed in their first year of life tend to show low IQ scores at four years of age [164]. The impact of the social environment on IQ is also testified by early childhood educational programs, which link emotional and motivational arousal with activities designed to exercise and promote selective attention or executive functions. These programs, indeed, have been proved to enhance various cognitive and school outcomes [165,166]. This supports the view according to which children extensively learn through social interactions with other people. The capacity to feel and regulate emotions is therefore another crucial aspect for understanding human intelligence [96].

### 4.6. Toward an Integrated Framework: Reaching a Dynamic Balance

In the first decades of life, enormous changes occur at the cellular, neural, behavioral and environmental levels. Despite the need of more research to disclose the interplay of these levels over developmental time and the specific mechanisms that prompt the construction of efficient mental representations (see for a discussion [167]), their influence on the development of intelligence cannot be questioned. In the previous paragraphs, we made an explicit attempt to describe how the different levels of constraints shape the development of human intelligence and, specifically, how they relate to neural representation and behavioral outcomes from a neuroconstructivist perspective.

**Figure 5:**
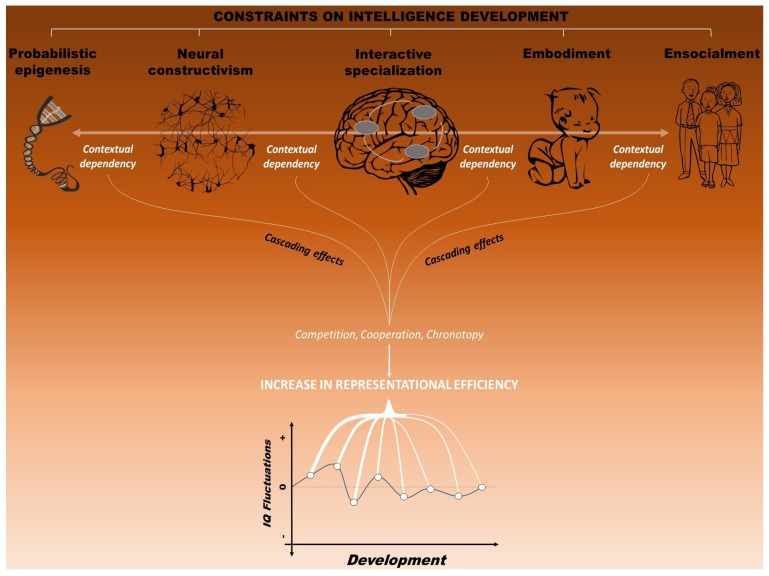
The multiple interacting constraints that influence the development of intelligence, conceived as the construction of efficient mental representation (i.e., neural activation patterns that sustain adaptive behavior). The principle of *context-dependence* constrains intellectual development by means of three general mechanisms: *cooperation*, *competition* and *chronotopy*. This last mechanism reflects the developmental essence of intelligence, as constraints are supposed to operate mainly during early phases of life, with intelligence profiles expected to be more stable in adulthood compared to infancy and early childhood. Together, all constraints imposed interactively by genes, brain, cognition, and environment are viewed as responsible for the fluctuations of intelligence over developmental time.

In particular, in our theoretical framework, intellectual development was conceived as a progressive increase in the efficiency of mental representations, defined as the neural activation patterns that sustain adaptive behavior in the environment. There is huge individual variability, indeed, in the way brains of different people adapt to the environment, with more intelligent individuals displaying more plastic brain and more efficient neural activations in a range of cognitive tasks [20]. Thus far, we have described how the interaction between multiple factors may lead to different levels of phenotypical performance in IQ tests. Indeed, the shaping of neural structures that allows the development of intelligence is dependent on the context in which the same structures develop (i.e., principle of *context dependence*) (see Figure 5). In this sense, all constraints imposed interactively by these different levels contribute to the dynamic equilibrium—characterized by alternations of stability and instability periods—supporting intellectual functioning over development [168].

However, how would intellectual development specifically progress? In line with the neuroconstructivist approach, we maintain that three domain-general, main mechanisms would guide the realization of more efficient representations over time: *competition*, *cooperation* and *chronotopy*. In particular, *competition* and *cooperation* would contribute to the increase in efficiency of the mental representations by allowing the specialization and the integration of functions, respectively. Indeed, this process of neuronal competition and gradual specialization over development would induce the brain to produce a more efficient activation for a certain kinds of input over others. The timing of these mechanisms is well captured by *chronotopy*, which refers to the temporal dynamic in which the *context-dependence* principle constrains the emergence of representations. Constraints are supposed to operate mainly during early phases of life, with intelligence profiles that are expected to be more stable in adulthood compared to infancy and early childhood, in line with the evidence reviewed above (see Figure 5). We further suggest that a positive *context-dependence* between different levels (i.e., genes, brain, cognition, and environment) may facilitate the functioning and the progress of the three mechanisms in more intelligent children. In particular, the fact that high intelligence is related to more plastic cortical development may indicate that the brain of these children adapt more efficiently to the requests from the environment.

### 4.7. Predictions and Directions for Future Research

The neuroconstructivist approach outlined above maintains that all of the constraints imposed by genes, brain, behavior, cognition and environment can simultaneously contribute to the dynamic equilibrium of intellectual functioning. Hence, a first core prediction of this model is that changes in IQ level should be expected over development and that these variations should be portioned out across the different levels of constraints.

A second prediction of this approach is that the weight of each constraint in influencing IQ should vary as a function of age. For this very reason, it is important to understand and investigate neural activation patterns that sustain adaptive behavior at multiple levels of analysis across development, ideally by means of longitudinal investigations. In analogy to transactional models of gene-environment correlation and to probabilistic epigenesis, we specifically hypothesize that the influence of genetic factors should be more variable in infancy and early childhood and become relatively stable only over time [168]. Indeed, gene-environment correlations may occur when children actively or passively seek out and choose experiences on the basis of their genetically influenced characteristics and motivations [168,169]. Accordingly, a positive feedback process may arise, with the child behaviors and motivations leading to experiences that reinforce those behaviors, which in turn lead the child to further engage in similar experiences [168,170]. Hence, not only are individuals with particular genotypes prone to select, evoke, and attend to particular environments and social contexts, but these environments are supposed to have causal, reciprocal influences on IQ [171]. Because these processes progressively accumulate over development, genetic effects should become more highly stable with time [168]. In analogy to the ensocialment level, the embodiment level is also supposed to exert its influence on cognitive abilities mainly during early stages of life.

Third, and in strict relationship with the previous prediction, because the chance to engage in particular experiences is dependent on the proactive exploration and manipulation of the environment, the development of the motor and attentional systems is hypothesized to mediate gene-environment correlations early in life. In particular, the rate of progression of infant motor development and early indices of attentional functioning are supposed to influence both the expression of genes relevant for cognitive abilities and the relative responses from the social environment.

Fourth, we expect that the neural architecture subserving intellectual functioning should be extensively shaped by the embodiment and the ensocialment levels. In particular, since the development of the attentional and sensory-motor systems is linked to individual differences in general cognitive abilities, we suggest that scores in these domains should be associated with total cerebral volume and with trajectories of cortical thinning. In similar terms, we expect a strong relationship between neural structures, the socioeconomic environment and IQ.

Fifth, we expect these levels to also impact on neural specialization. For instance, children who reach motor milestones earlier or who are more attentive should have brain networks that are more highly integrated at both global and local levels, with this association that should weaken as a function of age. Similarly, we expect that positive social contexts and educational programs should influence synaptic connectivity, gray matter thickness and myelination.

To sum up, we suggest that over developmental time intelligence can be subject to bidirectional interactions between gene activity, neural activity, behavior and the environment. Hence, at a higher level of our model, not only genes can prompt the level of efficiency of neural activations that, in turn, promote adaptive behavior, but this neural activation occurring in a developing brain may also lead to modifications in gene expression. In this regard, it is worth specifying that since our model is developmental, we do hypothesize a preferential direction between our levels of constraints, with genotypes shaping a series of endophenotypes (e.g., brain structure, white matter, etc.), which in turn would influence behavioral phenotypes (i.e., *gF*) (see for a proposal based on a hierarchical model [172]). However, we also hypothesize that these influences should become bidirectional over developmental time. In fact, the direct (i.e., bodily) experience in a certain environment and the social context in which the child is developing can further shape or mediate such a circular loop. Hence, it is from the mutual relationship between all these constraints that efficient neural representations develop and progress.

## 5. Cascading Effects on Life Outcome

A better understanding of the dynamics underlying intelligence development is fundamental not only from a clinical and scientific standpoint, but also for its impact on educational and public policy. Indeed, monitoring individual trajectories of IQ over time may bolster the child development into protected routes. This is especially relevant in light of the cascading effects that intellectual functioning exerts on many important life outcomes. For instance, individual differences in general intelligence are one of the strongest predictors of occupational attainment, social mobility [173] and job performance [174]. People with higher general intelligence in childhood or early adulthood also tend to have better overall physical health, and have longer life expectancy [175]. Further, children with higher childhood IQ have a lower risk of developing dementia [176] and of being diagnosed with schizophrenia spectrum disorder, major depression, or any anxiety disorder in adulthood [177]. Understanding how differential IQ levels become associated with these life outcomes is crucial not only from a scientific point of view, but also for developing public policy and ideating effective interventions.

The scientific significance of general intelligence and its potential for informing public policy is, however, mainly underappreciated [178]. We are, in fact, still a long way from taking advantage of scientific evidence to protect a child’s right to an open future. For instance, a large number of cognitive interventions have been shown to exert profound effects on both *g*F and *g*C and, more generally, on academic achievement, thus speaking against a predetermined trajectory of intelligence. In particular, there is clear evidence that formal education has a great impact on IQ [9,179]. When children are deprived of school for a protracted period of time they can exhibit deficits in IQ of as much as two standard deviations. Such effects of schooling on intelligence measures can be even twice the effect of age [180] and may depend on the age in which schooling begins [181]. Further, as reviewed above, targeted educational programs have the potential to promote various cognitive abilities [165,166]. However, despite the documented positive effects of schooling and educational programs on IQ, we still lack a precise identification of those variables that produce the greatest gains and, consequently, a direct connection between policy and research. In an ideal future, we should fulfill this gap and possibly provide teachers with specialized professional skills that help them cope with children with different IQ levels in a scientifically appropriate way [182].

Public policies should also subsidize a better communication between families whose children have intellectual disabilities, professionals involved in the diagnostic process, educators and teachers. In particular, all parties should be informed about the fluctuations that characterize intelligence across development. Viewing intelligence as a dynamic concept may indeed step up efforts toward practices for cognitive enhancement from professionals of the health care systems and from teachers. Similarly, families should be carefully informed about the positive effects that the social environment in which a child develops may exert on cognitive abilities.

## 6. Concluding Remarks

In recent times, intelligence research has profoundly advanced by integrating different fields of investigation. The interdisciplinary approach pursued by many recent studies has elegantly illustrated how genes, brain, cognition, and environment may interact with each other over development to influence intelligence profiles. This evidence indicates that intelligence is not static; rather, it undergoes extensive developmental changes over ontogenesis. In these terms, intelligence can be conceived as the phenotypic outcome arising from a fluctuating interaction between the developing system itself and the environment in which it progresses. Achieving this dynamic balance in intellectual skills is a milestone of ontogenetic development [183]. A vital issue for future consideration is the fact that we know that the microcircuitry of the brain develops enormously in the early postnatal life, followed by a period of pruning in which only used connection weights are strengthened [92]. However, we know very little about how the development of intelligence relates to this neural changes and to the progress toward representational efficiency. For instance, how is the development of specific intellectual skills (and the differentiation process between *g*F and *g*C) associated to the progressive modularization of the brain? How do genes specifically modify this relationship over time? What is the exact association between brain structure, representational efficiency and SES? Further, do the same neural and genetic aspects that contribute to intelligence later in life also contribute to attentive processes and the attainment of motor milestones in the first months of life? In short, there is an urgent need to longitudinally identify the developmental processes that cause the dynamic balance between the levels implied by the outlined neuroconstructivist approach through careful multidisciplinary investigations.

## Footnotes

1 Piaget was the first psychologist who theorized intelligence within a developmental framework. With admirable foresight, he postulated that intelligence would emerge from a dynamic interplay between the individual and the environment. Ironically, after decades of Nature–Nurture debate, we are still conceptualizing intelligence in similar terms. However, thanks to the recent emergence of the developmental cognitive neuroscience, the neuroepigenetics and the behavioral genetics fields, we can now more accurately define the strict interplay between the developing organism and the environment, which are viewed as active entities exerting cascading effects on intellectual functioning.
